# Living with own or husband's mother in the household is associated with lower number of children: a cross-cultural analysis

**DOI:** 10.1098/rsos.170544

**Published:** 2017-10-25

**Authors:** Susanne Huber, Patricia Zahourek, Martin Fieder

**Affiliations:** Department of Anthropology, University of Vienna, Vienna, Austria

**Keywords:** mother, mother in law, kin support, female dispersal, number of grandchildren

## Abstract

It has been discussed in the literature that the presence of kin, particularly the presence of a women's own mother or her mother in law, may have fertility effects. We aimed to examine the effects of the presence of a woman's own or her husband's mother in the household on a woman's fertility in terms of number of children on a broad basis by analysing census data of over two million married women aged between 15 and 34 years from 14 countries worldwide. We find that with the exception of Iraq, across all countries, the majority of women live only with their spouse in the household. We further find that the presence of any mother in the household is invariably associated with a significantly lower number of children compared to women living only with their spouse. In addition, in most countries, a woman's number of children is lower if she lives with her own mother as compared to her husband's mother in the household. Number of children is nonlinearly associated with woman's age, the presence of any mother being related with an earlier start of childbearing but a shallower increase in number of children. We speculate that the presence of a mother in the household may slow down woman's reproduction, but also discuss alternative explanations.

## 1. Introduction

In humans, the long period of dependency of children, the simultaneous rearing of children and the short intervals between births mean a high demand of time and energy particularly for the mother. Kaplan [1] calculated that a child consumes from birth to age of 18 years a surplus of 10–13 million calories. It is thus widely agreed that help for the mother increases her reproductive output and the survival of her children [2]. In line with this, Hrdy [3,4] formulated the cooperative breeding hypothesis: mothers have evolved in groups where not only the biological father but a variety of individuals provided resources for the children. According to Hamilton's rule, these support-providing individuals are expected to be close kin [3,5,6] who can increase their indirect biological fitness by helping their kin to reproduce. Hence, several relatives, apart from the parents, may invest in the child, depending in part on their relatedness to the child: the closer the kinship, the higher the investment. Here, in particular grandparents in their post-reproductive period may take the chance to increase indirect fitness by helping their own offspring to reproduce and investing in their grandchildren [7], with whom they are genetically related to an average 25%. In addition to promoting grandchildren survival, grandparents also foster infant mental development [8] and are protective for their grandchildren (rev. in [9]) although data from Switzerland show that the investment of grandparents seems to decrease with increasing number of grandchildren [10].

Especially grandmothers play relevant roles in the lives of their grand-offspring, whereas the contribution of fathers and grandfathers to child survival, if existing, is relatively small [2], although a study by Sear & Coall [11] found that the presence of the maternal or the paternal father may have a pro-fertile effect. In humans, women represent a ‘biological anomaly’ as they can live substantial time after the termination of reproduction [12]. According to Williams [12] this may be adaptive if such a premature end of reproduction benefits the woman's survival, for instance, because she would not face the danger of dying during childbirth (rev. in [13,14]), so that she can devote her full parenting effort to her already born children who are dependent upon the mother for several years. In addition, in this way she can also invest in her offspring's reproduction and improve survival of her grandchildren. Correspondingly, by analysing multi-generational demographic records from Finland and Canada, Lahdenpera *et al*. [15] showed that women living longer post-reproductively have more grandchildren. It has, however, also been argued that the post-productive lifespan may not be adaptive (rev. in [9]).

Nonetheless, in his review, Euler [16] comes to the overall conclusion that in pre-modern, natural fertility populations, grandmothers increase the probability of child-survival, a pattern that can also be found in modern societies among families living in high risk circumstances. Whether effects differ between maternal and paternal grandmothers, however, is less clear. Evidence from natural fertility societies (rev. in [2]) suggests that the presence of both the maternal (9 of 13 studies) or the paternal grandmother (9 of 17 studies) is associated with increased chances of child survival. Although some studies point to positive impacts of paternal grandmothers on survival of their grandchildren [17,18], other studies find that the presence of paternal grandmothers increases the risk of stillbirths and infant mortality [19,20]. Also, a study on Oromo grandmothers in rural Ethiopia [21] reports that children born in a matrilocal household have a significantly lower risk of dying than those born in a patrilocal residence.

Compared to their effect on child survival, the effects of both maternal and paternal grandmother's presence on fertility are less well understood. Most of the research on maternal grandmothers reports a positive impact on their daughter's fertility [2]. In a meta-analysis, Sear & Coall [11], on the other hand, found that only in over 30% of 39 reviewed studies the presence of the own mother, but in approximately 70% of the studies, the presence of the husband's mother has a pro-natal effect. Hence, there is need for an examination of the effects of maternal and paternal grandmother's presence on a broader basis. We therefore performed a cross-sectional analysis of 14 countries from different parts of the world. These countries include different ethnicities, cultures, and economic developments, offering at least to some extent the potential to overcome a fundamental critique of Henrich *et al*. [22], namely that the variation across human population in many studies is so small that samples based on individuals from Western, Educated and Industrialized and Democratic (WEIRD) societies are taken as representative for the species. As, however, obviously culture, habits and economics are very different in different populations across the planet, this view may not hold true. We thus included each country in our analysis for which data were available, investigating if and how the absence or presence in the household of a woman's own or her husband's mother is associated with the woman's number of biological children. This ended up with 14 census samples covering a wide range of geographical, ethnic, cultural and economic varieties, which allows to find effects that are potentially common in most contemporary human populations as well as effects that are unique to a specific country.

## 2. Material and methods

We used the census data of 2 478 383 married women aged between 15 and 34 years from 14 countries provided by IPUMS international (https://international.ipums.org/international/) (table 1). This age range means that the women are in their reproductive years and thus have not completed reproduction. Despite this limitation, we had to use this age range as we were interested in the effects of the presence of a woman's own mother or her mother-in law in the household and many of those would already have died if we had analysed women in their post-reproductive years.

**Table 1.** Number of cases and percentages where either no mother, the woman's own mother, or her husband's mother is present in the household. HH, household.

	no mother in HH		own mother in HH		mother in law in HH		total *N*
Argentina 2001	76 003	85.84%	5336	6.03%	7203	8.14%	88 542
Brazil 1991	470 618	88.42%	28 184	5.30%	33 450	6.28%	532 252
Greece 2001	24 652	87.10%	774	2.73%	2878	10.17%	28 304
Indonesia 1990	47 008	77.21%	5706	9.37%	8166	13.41%	60 880
Iraq 1997	48 438	45.67%	1246	1.17%	56 371	53.15%	106 055
Malawi 2008	90 044	96.27%	736	0.79%	2755	2.95%	93 535
Malaysia 1980	6812	73.84%	526	5.70%	1887	20.46%	9225
Pakistan 1973	39 262	57.67%	764	1.12%	28 060	41.21%	68 086
Philippines 1990	315 127	85.34%	22 330	6.05%	31 821	8.62%	369 278
Romania 2002	76 582	69.69%	10 662	9.70%	22 648	20.61%	109 892
Sudan 2008	189 737	91.18%	2986	1.43%	15 364	7.38%	208 087
Thailand 1980	17 999	68.89%	4481	17.15%	3649	13.97%	26 129
United States 1980	692 733	97.11%	10 089	1.41%	10 519	1.47%	713 341
Zambia 2010	61 860	95.50%	751	1.16%	2166	3.34%	64 777
Sum	2 156 875	87.02%	94 571	3.82%	226 937	9.16%	2 478 383

To avoid redundancies we included only the most recent census sample of a country for which the needed information is available. The sample identifier (sample number) provided by IPUMS international is unique for the country and the year of the census. We included this unique sample identifier as mixed factor in our multivariate models to account for country and year of census. The fraction of the population that has been sampled differs from census to census, ranging from 0.51% to 10% of the total population of a country. Also the population sampled differs among censuses. A detailed description of the population sampled for each country and the sampling strategy for each country can be found in electronic supplementary material, tables S1–S6.

We only included women who are currently married and where their spouse is present in the household. The characteristics of the spouse and the woman's mother have been linked by IPUMS international. In addition, we linked the characteristics of the husband's mother via the location of the spouse's mother in his household, which has also been provided by IPUMS international.

We included the following variables in the analysis: number of children the women has born until the time of the census (we used this parameter as dependent variable as it is well kept in international census databases and, thus, available for all datasets, whereas including child mortality as in many other studies, would have led to a very limited number of cases). We further included the woman's age; an estimate for the woman's reproductive time period calculated as the difference in years between current age at the time of the census and age at the woman's first marriage, which we used as proxy for reproductive onset because data on age at first birth are not provided; presence of the woman's or her husband's mother in household encoded as 0 = no mother present, 1 = the woman's own mother present, and 2 = her husband's mother present (as the case, that both mothers are present in a household was rare (460 cases), these cases were excluded from the analyses); the woman's and her spouse's highest educational attainment, encoded as 1 = less than primary completed, 2 = primary school completed, 3 = secondary completed, 4 = university completed; the woman's and her spouse's current employment encoded as 0 = not employed, and 1 = currently employed; as well as living area encoded as 1 = rural, and 2 = urban. In addition, we included whether or not the woman's or her husband's mother living in the household are still in their reproductive years, encoded as 1 = the woman's or her husband's mother, respectively, is younger than 46 years, and 2 = if she is at least 46 years at the time of the census.

We used SPSS 21 and R 3.3.1 (libraries MASS and mgcv) for all analyses. On basis of these variables, we calculated a generalized linear model including all available variables (‘full model’ see below) regressing on the woman's number of children, on basis of a Poisson error structure with sample as random factor, using the function glmPQL provided by the MASS library in R. In the electronic supplementary material (tables S7–S11), we additionally provide the results of stepwise elaborating generalized linear models, starting with a first, most simple model, only including the woman's age and reproductive time period, as well as if the woman's own or her husband's mother is present in the household, regressing on the woman's number of children, on basis of a Poisson error structure, with sample as random factor. In the successive models, we further added (i) the woman's education, (ii) the woman's employment, (iii) her spouse's education, and (iv) her spouse's employment. Additional inclusion of living area represents the ‘full model’ presented in the results. Moreover, we calculated a separate ‘full’ generalized linear model for each census (electronic supplementary material, table S12). We set ‘own mother in household’ as reference in all samples, as women living with their own mothers had, on average, the lowest number of children. So, if the category ‘no mother present’ or ‘husband's mother present’ significantly differs from the reference, this usually means a higher number of offspring (i.e. the sign is positive).

In addition to the full generalized linear mixed model, we also calculated a generalized additive mixed model (function bam in the R mgcv library) to investigate potential nonlinear effects of age with respect to the presence of the woman's own or her husband's mother in the household, including all variables of the full model, and plotted the smoothed curves of the association between the woman's age and her number of children according to the presence of own, husband's or no mother in the household. We further investigated which variables predicted co-residence with either the woman's or her spouse's mother, applying a generalized linear mixed model with sample as random factor.

## 3. Results

In all but one country, the highest percentage of women live in households without any mother present. The percentage of women living with their own mother range from 0.79% (Malawi) to 17.15% (Thailand), that of women living with their husband's mother ranges from 1.47% (USA) to 53.15% (Iraq) (table 1).

We find that regardless of statistical model (tables 2–5; electronic supplementary material, tables S7–S11), the absence of any mother in the household has a significant positive effect on a woman's number of children: in all models, women living without any mother in the household have significantly more children than those living with their own mother, and—as indicated by lower estimates—also more children than those living with their husband's mother in the household. Note that the woman's spouse is always present in the household. In the case that a mother is present in the household, it is less clear whether a woman has more children if this is her own or her husband's mother (tables 2–5; electronic supplementary material, tables S7–S11): signs and significances vary both among differently complex models (table 2) as well as among different countries (table 3). Only in the most simple model (electronic supplementary material, table S7), compared to the presence of a woman's own mother, the presence of the husband's mother has a significant positive effect on the woman's number of children. As soon as woman's education is added to the model, however, sign and/or significance levels change, varying among different models from non-significantly positive (‘full model’, table 4) to significantly negative (electronic supplementary material, tables S9 and S11). Also, the separate models for each census differ as regards the relative effects of the presence of either the woman's own or her husband's mother on her offspring number—again the most children are invariably found in women living without any mother—although in most countries, it is the presence of the husband's mother that has a more positive effect (table 3): in most countries, a woman's number of children is higher if the husband's as compared to her own mother is present in the household, the difference being significant in seven countries. Only in two countries, women have significantly more offspring if her own as compared to her husband's mother is present in the household.

**Table 2.** Sign and significance of estimates obtained from increasingly complex generalized linear mixed models (complete models see electronic supplementary material, tables S7–S11 for models 1–5, and table 4 for full model). Italic: significant positive effect on woman's number of children; bold: significant negative effect on woman's number of children; underline: not significant. HH, household.

	Model 1	Model 2	Model 3	Model 4	Model 5	full model
no mother in HH (ref.: own mother in HH)	*0*.135^***^	*0*.111^***^	*0*.105^***^	*0*.11^***^	*0*.103^***^	*0*.1231^***^
husband's mother in HH (ref.: own mother in HH)	*0*.022^***^	−0.001	−**0**.008*	−0.006	−**0**.0061*	0.0023
woman's age	*0*.007^***^	*0*.02^***^	*0*.021^***^	*0*.022^***^	*0*.022^***^	*0*.0229^***^
woman's reproductive time span	*0*.11^***^	*0*.1^***^	*0*.094^***^	*0*.093^***^	*0*.094^***^	*0*.093^***^
woman: education primary completed (ref.: less than primary completed)		−**0**.1^***^	−**0**.094^***^	−**0**.067^***^	−**0**.067^***^	−**0**.0728^***^
woman: education secondary completed (ref.: less than primary completed)		−**0**.291^***^	−**0**.262^***^	−**0**.2^***^	−**0**.2^***^	−**0**.1836^***^
woman: university completed (ref.: less than primary completed)		−**0**.556^***^	−0493^***^	−**0**.38^***^	−**0**.38^***^	−**0**.2776^***^
woman: employed (ref.: not employed)			−**0**.188^***^	−**0**.19^***^	−**0**.19^***^	−**0**.106^***^
spouse: education (ref.: less than primary completed)				−**0**.041^**^	−**0**.041^**^	−**0**.0409^***^
spouse: education secondary completed (ref.: less than primary completed)				−**0**.092^***^	−**0**.093^***^	−**0**.073^***^
spouse: university completed (ref.: less than primary completed)				−**0**.195^***^	−**0**.194^***^	−**0**.1087^***^
spouse: employed (ref.: not employed)					*0*.0292^***^	*0*.039^***^
urban (ref.: rural)						−**0**.0638^***^

*p < 0.05.

**p < 0.01.

^***^p < 0.001.

**Table 3.** Estimates and significance for presence of no mother and husband's mother in the household, respectively, as compared to presence of a woman's own mother in the household, obtained by separate generalized linear mixed models for each census, of woman's age, her reproductive time span, woman's and her spouse's education and employment, living area, and presence of any mother in the household, regressing on the woman's number of children on basis of a Poisson error structure, with sample as random factor (full models for each census separately see electronic supplementary material, tables S12). Italic: significant positive effect on woman's number of children; bold: significant negative effect on woman's number of children; underline: not significant.

	no mother in household (ref.: own mother in household)	husband's mother in household (ref.: own mother in household)
Argentina	*0*.1150^***^	*0*.0421^**^
Brazil	*0*.0512^***^	−**0**.0871^***^
Greece	*0*.0726*	*0*.1850^***^
Indonesia	*0*.2740^***^	*0*.2211^***^
Malawi	*0*.2004^***^	0.0592
Iraq	*0*.2099^***^	*0*.1216^***^
Malaysia	*0*.0840*	0.0791
Pakistan	*0*.1600^***^	0.0150
Romania	*0*.1117^***^	*0*.0409^***^
USA	*0*.0325^***^	0.0083
Thailand	*0*.1796^***^	*0*.1122^***^
Sudan	*0*.3919^***^	*0*.2246^***^
Philippines	*0*.1886^***^	*0*.0190*
Zambia	*0*.0554*	−**0**.0607*

*p < 0.05.

**p < 0.01.

***p < 0.001.

**Table 4.** Generalized linear mixed model (‘full model’) of woman's age, her reproductive time span, woman's and her spouse's education and employment, living area, and presence of any mother in the household, regressing on the woman's number of children on basis of a Poisson error structure, with sample as random factor. HH, household.

	value	s.e.	*t*-value	*p*-value
(intercept)	−0.5496	0.0591	−9.3057	*p* < 0.0001
no mother in HH (ref.: own mother in HH)	0.1231	0.0028	43.4389	*p* < 0.0001
husband's mother in HH (ref.: own mother in HH)	0.0023	0.0032	0.7014	*p* = 0.4831
woman's age	0.0229	0.0002	135.4527	*p* < 0.0001
woman's reproductive time span	0.0930	0.0002	565.0516	*p* < 0.0001
woman: primary school completed (ref.: less than primary completed)	−0.0728	0.0014	−52.2709	*p* < 0.0001
woman: secondary completed (ref.: less than primary completed)	−0.1836	0.0020	−93.5303	*p* < 0.0001
woman: university completed (ref.: less than primary completed)	−0.2776	0.0035	−78.6854	*p* < 0.0001
spouse: primary school completed (ref.: less than primary completed)	−0.0409	0.0014	−30.1645	*p* < 0.0001
spouse: secondary completed (ref.: less than primary completed)	−0.0730	0.0018	−40.2508	*p* < 0.0001
spouse: university completed (ref.: less than primary completed)	−0.1087	0.0032	−34.0977	*p* < 0.0001
woman: employed (ref.: not employed)	−0.1060	0.0012	−87.1489	*p* < 0.0001
spouse: employed (ref.: not employed)	0.0390	0.0017	23.2479	*p* < 0.0001
urban (ref.: rural)	−0.0638	0.0012	−54.7132	*p* < 0.0001
DF	1522705			
	(intercept)	residual		
SAMPLE StdDev:	0.1861625	0.8286739		
residual deviance: 1284447 on 1522714 degrees of freedom				
AIC: 4608709

**Table 5.** Generalized additive model of woman's age, her reproductive time span, woman's and her spouse's education and employment, living area and presence of any mother in the household, regressing on the woman's number of children on basis of a Poisson error structure, with sample as random factor.

	estimate	s.e	*z*-value	*p*-value
(intercept)	−0.0443	0.0613	−0.7230	*p* = 0.643
woman's reproductive time span	0.0940	0.0002	471.6250	*p* < 0.0001
woman: primary school completed (ref.: less than primary completed)	−0.0819	0.0017	−48.7480	*p* < 0.0001
woman: secondary completed (ref.: less than primary completed)	−0.1972	0.0024	−83.3950	*p* < 0.0001
woman: university completed (ref.: less than primary completed)	−0.2808	0.0043	−66.0430	*p* < 0.0001
spouse: primary school completed (ref.: less than primary completed)	−0.0435	0.0016	−26.5860	*p* < 0.0001
spouse: secondary completed (ref.: less than primary completed)	−0.0783	0.0022	−35.8190	*p* < 0.0001
spouse: university completed (ref.: less than primary completed)	−0.1105	0.0038	−28.7230	*p* < 0.0001
woman: employed (ref.: not employed)	−0.1020	0.0015	−69.5610	*p* < 0.0001
spouse: employed (ref.: not employed)	0.0356	0.0020	17.6190	*p* < 0.0001
urban (ref.: rural)	−0.0587	0.0014	−41.7100	*p* < 0.0001

approximate significance of smooth terms:				
	edf	Ref.df	Chi.sq	*p*-value
s(Age): *no mother in the household*	8.952	8.999	46 898	*p* < 0.0001
s(Age): *woman's own mother in household*	7.013	8.037	3227	*p* < 0.0001
s(Age): *her husband's mother in household*	8.693	8.968	15 241	*p* < 0.0001
s(SAMPLE)	8.997	10	68 222	*p* < 0.0001
deviance explained = 42%				
fREML = 1983400				
AIC = 4490739				

Apart from the effects of the absence or presence of any mother in the household, in the ‘full’ generalized linear mixed model (table 4), a woman's age and her reproductive time span (as women are still in their reproductive years), as well as her spouse's employment are significantly positively associated with her number of children, whereas the woman's own employment and higher education of both the woman and her spouse have a significant negative effect on the woman's offspring number. Also, living in an urban area is associated with lower woman's number of children.

**Figure 1.**
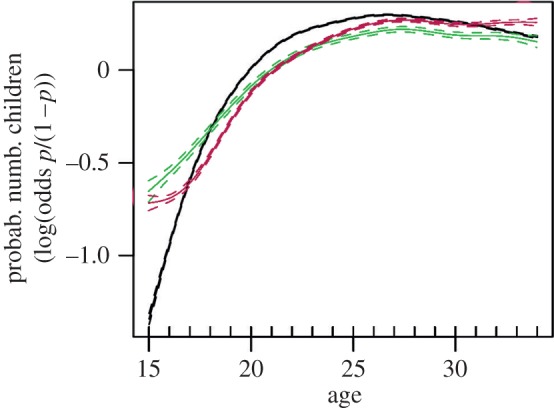
Smoothed curves of woman's age (*x* axes on a linear scale) and the probability for a certain number of children for this age (*y*-axis in logits (log (odds *p*/(1 − *p*)), i.e. the values are centred on 0 (50/50 odds)) if no mother is present in the household (black curve); woman's own mother is present in the household (green curve); or husband's mother is present in the household (red curve).

Although signs and significances of the full generalized linear mixed model are comparable with those of a generalized additive mixed model (c.f. table 4 and table 5), the smoothed curves reveal a nonlinear relationship between a woman's age and her number of children, which differs with respect to the absence or presence of any mother in the household (figure 1): in the case that no mother is present in the household, at the age of 15 years, women are usually childless, with a steep increase in number of children until the age of approximately 20 years and a maximum at the age of approximately 27 years. In contrast, both women living with their own or their husband's mother in the household start with a higher average number of children already at age 15 years as compared to women living without any mother in the household. The further increase in number of children with increasing age, however, is less steep and also reaches a maximum at about 27 years of age (figure 1).

Investigating which variables predicted living with a woman's mother in the household, we find that both the woman's and partially also her spouse's increasing education, the woman's employment as well as living in an urban area are significantly positively associated, whereas woman's age and spouse's employment are significantly negatively associated with living with the woman's mother in the household (electronic supplementary material, table S13a). Living with the spouse's mother in the household is predicted both by increasing woman's and her spouse's education and woman's employment. Woman's age, living in an urban area and spouse's employment, on the other hand, are significantly negatively associated with living with spouse's mother in the household (electronic supplementary material, table S13b).

## 4. Discussion

Across all countries, the majority of women live only with their spouse in the household. Only in Iraq, the majority of couples live with the husband's mother in the household (53.15%), followed by Pakistan (41.2%). In all other investigated countries, the frequency of living with husband's mother in the household ranges roughly between 1.5% (USA) and 20.5% (Romania). Living with the woman's own mother in the household is the least common living form, frequencies ranging from 0.8% (Malawi) up to 17.2% (Thailand). In addition, the negative association between woman's age and living with any mother in the household indicates that younger women are more likely to live with any mother in the household than older ones. As our data are from recent societies, however, they provide no evidence whether living alone as a couple has always been the most common living arrangement. Maybe, living alone as a couple is only the result of more recent social and economic developments. Nonetheless, our data do not support the claim of patrilocality—which means that women move to their spouse's household to live there in co-residence with their husbands and their husband's kin [23]—for the age group investigated, even though it has been claimed that humans generally favour patrilocal female dispersal [24].

The presence of either a woman's own or her husband's mother in the household is invariably associated with a significantly lower number of children compared to women living only with their spouse. Although the smoothed curves indicate that childbirth starts at an earlier age in women living with either their own or their husband's mother in the household, the increase in number of children with increasing age is much steeper in women living without any mother. Our data thus point to lower fertility in the case of the presence of any mother in the household. This is interesting, as one would expect that both mothers should have ‘some biological interest’ in a higher number of grandchildren.

Our finding of lower fertility whenever any mother is present in the household is in conflict with studies reporting on a pro-natal effect (see below). Possibly, effects differ between developing and developed countries. By far the most censuses included in our study are from developing countries, whereas the following studies pointing to a pro-natal effect of the presence of any grandmother are from developed countries. In Japan, for instance, the presence of paternal mothers have a strong positive effect on fertility [25]. Kaptijn *et al*. [26] found for The Netherlands that support from grandparents in general increased the probability of parents having additional children within a timeframe of 8–10 years. Also in a British sample, the presence of grandparents helped to increase reproduction [27]. Yet, another study shows a more differentiated pattern for Great Britain: contact with paternal grandparents increases the chances of having a second child but contact with the maternal grandparents decreases the chance of a third or higher order child [28].

Apart from our overall finding of lower fertility in the presence of any mother in the household, this effect seems to be more pronounced if it is the woman's own as compared to the husband's mother present in the household: in most but not all censuses, a woman's number of children is higher if this is the husband's as compared to the woman's own mother. Only in two censuses, fertility is lower if the husband's mother as compared to the woman's own is present in the household. Also Sear & Coall [11] report that the presence of paternal mothers have a pro-natal effect in more studies than the presence of maternal mothers. This finding could be interpreted in the light of paternity uncertainty [29,30]. According to Kemkes-Grottenthaler [17], the husband's mother focuses her attention on her daughter-in-law, thereby functioning as ‘watchful instances’, decreasing the probability of cuckoldry and reducing paternity uncertainty of her son, which would be associated with decreased investment [29]. In addition, as husband's mothers are genetically related to their grandchildren but not to their daughters-in-law, they might have an interest to exploit the woman reproductively [31]. The husband's mother may also provide support for her daughter-in-law, which may increase probability of pregnancy. On the other hand, moving to her spouse's household may expose a woman to the risk of decreased access to resources, which may decrease her fertility.

Generally, our results can also be viewed in the light of local resource competition, emphasizing the competition for resources among family members. In three-generation households, grandparents are not only providers of support but can also be resource competitors. Support for the resource competition theory comes from a meta-analysis of 17 studies by Strassmann & Garrard [32] on the association between grandparental and grandchild survival in patrilineal populations that mostly found that living with grandparents has a less beneficial association for the grandchildren than not living with grandparents. Also Strassmann [33] found in the Dogon, that the risk of death for a child was twofold higher if the co-resident parental grandmother was alive. She argued that in this resource-poor society, elderly grandmothers become more net-consumers than net-producers. We did not analyse child survival. Nonetheless, to some extent this is also in line with our finding of lower fertility if any grandmother is present in the household.

Our data show an earlier start of childbearing but shallower increase in number of children if any mother is present in the household. One could speculate that both the woman's own as well as her husband's mother's presence may slow down the woman's reproduction. Hence both grandmothers may foster a ‘slower life-history strategy’ in terms of investing more in a smaller number of children. Indeed, the presence of a grandmother in the household usually increases survival of her grandchildren (rev. [2,16]), albeit the presence of a paternal grandmother has also been shown to have harmful effects [2,21,34]. An alternative explanation would be that the presence of any mother in the household does not affect a woman's number of children, but that women who start childbirth at very young ages have both a higher probability to live with a mother in the household and to have lower overall fertility. A possible reason for such a putative association could be that the presence of any mother in the household may be associated with socio-economic conditions that in turn may affect fertility. For instance, women living with any mother in the household might face a difficult and complicated stage of life (e.g. poor health, unemployment, etc.). Also, in the case that the husband moves to his wife's family, it could be argued that he may not be able to provide the necessary resources and this may lead to a decrease of fertility of such a ‘family constellation’. Accordingly, it has been shown that spouse's income affects a woman's fertility in a US sample [35].

This view is supported by a negative association between spousal but not woman's own employment and the presence of any mother in the household. We may speculate that living with any mother in the household may at least partially be a question of male status, and that unemployed men may have to live with any mother due to economic reasons. Unemployment may also to some extent explain the lower number of children as it is well known that lower male social status is associated with a lower offspring number [36–40]; though woman's employment and both woman's and spouse's education (an indicator for potential status [41,42]), on the other hand, increases the chance of living with any mother in the household.

### 4.1. Limitation of the study

We were not able to restrict our sample to women who have already completed reproduction, as in these women (older than 45 years) a considerable proportion of mothers would already have died because only in 5 of the 14 analysed censuses, life expectancy at the time of the census is higher than 70 years, and in 3 censuses, it is even lower than 60 years. Also, current marriage may not be the first marriage. Hence, husband's mothers may not be the grandmothers of all children.

## Supplementary Material

Supplementary tables

## Data accessibility

Data supporting this article are available at https://international.ipums.org/international-action/variables/RELIGION#codes_section and https://international.ipums.org/international-action/variables/RACE#codes_section.
