# Supplementary material for: Living with own or husband's mother in the household is associated with lower number of children: a cross-cultural analysis
Source: R Soc Open Sci. 2017 Oct 25;4(10):170544. doi: 10.1098/rsos.170544 (PMC5666251; doi:10.1098/rsos.170544)
Supplement: Supplementary tables [file rsos170544supp1.docx]

**Supplement**

**Table S1**. Women’s education in percentage for each census.

|  | Less than primary completed | | Primary completed | | Secondary completed | | University completed | |
| --- | --- | --- | --- | --- | --- | --- | --- | --- |
| Argentina 2001 | 3885 | 4.40% | 38679 | 43.70% | 39223 | 44.30% | 6755 | 7.60% |
| Brazil 1991 | 293047 | 55.10% | 143510 | 27.00% | 78661 | 14.80% | 17034 | 3.20% |
| Greece 2001 | 755 | 2.70% | 8111 | 28.70% | 13835 | 48.90% | 5603 | 19.80% |
| Indonesia 1990 | 23199 | 38.10% | 29692 | 48.80% | 7534 | 12.40% | 452 | 0.70% |
| Iraq 1997 | 44334 | 41.80% | 48298 | 45.60% | 10379 | 9.80% | 3016 | 2.80% |
| Malawi 2008 | 64417 | 69.20% | 22790 | 24.50% | 5782 | 6.20% | 104 | 0.10% |
| Malaysia 1980 | 2845 | 30.80% | 6109 | 66.20% | 152 | 1.60% | 119 | 1.30% |
| Pakistan 1973 | 59989 | 88.10% | 6949 | 10.20% | 673 | 1.00% | 469 | 0.70% |
| Philippines 1990 | 66129 | 18.20% | 148108 | 40.70% | 116135 | 31.90% | 33837 | 9.30% |
| Romania 2002 | 4116 | 3.70% | 30677 | 27.90% | 65639 | 59.80% | 9419 | 8.60% |
| Sudan 2008 | 184183 | 91.40% | 13959 | 6.90% | 661 | 0.30% | 2768 | 1.40% |
| Thailand 1980 | 22992 | 88.30% | 1942 | 7.50% | 938 | 3.60% | 164 | 0.60% |
| United States 1980 | 6570 | 0.90% | 115546 | 16.20% | 479828 | 67.30% | 111397 | 15.60% |
| Zambia 2010 | 25781 | 39.80% | 31636 | 48.80% | 7185 | 11.10% | 175 | 0.30% |

**Table S2**. Women’s mean age of marriage for each census.

|  | **Mean** | **Std. Dev** |
| --- | --- | --- |
| Argentina 2001 | 21.50 | 3.86 |
| Brazil 1991 | 19.75 | 3.73 |
| Greece 2001 | 21.65 | 3.90 |
| Indonesia 1990 | 18.36 | 3.12 |
| Iraq 1997 | 19.56 | 3.75 |
| Malawi 2008 | 18.14 | 2.47 |
| Malaysia 1980 | 20.27 | 3.37 |
| Pakistan 1973 | 17.33 | 2.53 |
| Philippines 1990 | 20.15 | 3.43 |
| Romania 2002 | 20.61 | 3.12 |
| Sudan 2008 | 17.35 | 3.37 |
| Thailand 1980 | 19.29 | 3.01 |
| United States 1980 | 20.12 | 2.92 |
| Zambia 2010 | 18.36 | 3.01 |

**Table S3**. Percentage of employed and unemployed women and husbands, respectively, for each census.

|  | Woman un-employed | | Woman employed | | Husband un-employed | | Husband employed | |
| --- | --- | --- | --- | --- | --- | --- | --- | --- |
| Argentina 2001 | 57898 | 65.40% | 30644 | 34.60% | 15166 | 17.10% | 73376 | 82.90% |
| Brazil 1991 | 379172 | 71.20% | 153080 | 28.80% | 15027 | 2.80% | 517225 | 97.20% |
| Greece 2001 | 14703 | 51.90% | 13601 | 48.10% | 2116 | 7.50% | 26188 | 92.50% |
| Indonesia 1990 | 37854 | 62.20% | 23026 | 37.80% | 1269 | 2.10% | 59611 | 97.90% |
| Iraq 1997 | 99409 | 93.80% | 6561 | 6.20% | 12822 | 12.10% | 92974 | 87.90% |
| Malawi 2008 | 45273 | 48.40% | 48262 | 51.60% | 15970 | 17.10% | 77565 | 82.90% |
| Malaysia 1980 | 6218 | 68.50% | 2862 | 31.50% | 198 | 2.20% | 9010 | 97.80% |
| Pakistan 1973 | 65669 | 96.50% | 2417 | 3.50% | 5180 | 7.60% | 62906 | 92.40% |
| Philippines 1990 | 278118 | 75.30% | 91160 | 24.70% | 51599 | 14.00% | 317679 | 86.00% |
| Romania 2002 | 45929 | 41.80% | 63963 | 58.20% | 18330 | 16.70% | 91562 | 83.30% |
| Sudan 2008 | 174425 | 83.80% | 33662 | 16.20% | 30542 | 14.70% | 177545 | 85.30% |
| United States 1980 | 325145 | 45.60% | 387597 | 54.40% | 58493 | 8.20% | 654751 | 91.80% |
| Zambia 2010 | 36534 | 56.40% | 28243 | 43.60% | 8525 | 13.20% | 56252 | 86.80% |

**Table S4**. Mean number of children born for each census

|  | **Mean** | **Std. Dev** |
| --- | --- | --- |
| Argentina 2001 | 1.80 | 1.37 |
| Brazil 1991 | 1.80 | 1.47 |
| Greece 2001 | 1.13 | 0.93 |
| Indonesia 1990 | 1.77 | 1.39 |
| Iraq 1997 | 2.37 | 1.87 |
| Malawi 2008 | 2.15 | 1.50 |
| Malaysia 1980 | 2.04 | 1.49 |
| Pakistan 1973 | 1.95 | 1.81 |
| Philippines 1990 | 2.16 | 1.56 |
| Romania 2002 | 1.17 | 0.92 |
| Sudan 2008 | 1.98 | 1.73 |
| Thailand 1980 | 1.90 | 1.45 |
| United States 1980 | 1.33 | 1.17 |
| Zambia 2010 | 2.29 | 1.70 |

**Table S5**. Mean number of children and mother’s location in the household for each census. Red numbers indicate lowest number mean number of children; green numbers indicate highest mean number of children among the three variables.

|  | **no mother in HH** | **own mother in HH** | **mother in law in HH** |
| --- | --- | --- | --- |
| Argentina 2001 | 1.85 | 1.38 | 1.56 |
| Brazil 1991 | 1.87 | 1.43 | 1.24 |
| Greece 2001 | 1.12 | 0.99 | 1.25 |
| Indonesia 1990 | 1.90 | 0.97 | 1.57 |
| Iraq 1997 | 2.88 | 1.67 | 1.95 |
| Malawi 2008 | 2.17 | 1.66 | 1.61 |
| Malaysia 1980 | 2.15 | 1.55 | 1.79 |
| Pakistan 1973 | 2.28 | 1.40 | 1.52 |
| Philippines 1990 | 2.31 | 1.26 | 1.36 |
| Romania 2002 | 1.21 | 0.99 | 1.08 |
| Sudan 2008 | 2.04 | 0.85 | 1.42 |
| Thailand 1980 | 2.07 | 1.29 | 1.63 |
| United States 1980 | 1.34 | 1.19 | 1.18 |
| Zambia 2010 | 2.31 | 2.02 | 1.69 |

**Table S6**.

|  | **Population as described at IPUMS international** | **Fraction of the Population** | **Sampling** | **Futher Information** |
| --- | --- | --- | --- | --- |
| Argentina 2001 | All the population in the national territory at the moment the census is carried out. This includes everyone (of any nationality) that spent the night of November 16-17 in any Argentine diplomatic embassy abroad; all the sailors or fishermen that spent the night of November 16-17 in ships with an Agentine flag or a foreign one docked in Argentine waters; and all Argetine workers that are abroad performing missions for the national government. | 10% sample | Systematic sample of every 10th private household and collective quarters with a random start. The sample was drawn by INDEC from the microdata of 100 percent of households. | https://international.ipums.org/international-action/sample_details/country/ar#ar2001a |
| Brazil 1991 | People who spent the night of August 31st to September 1st, 1960, as well as the people who were temporarily absent from the dwelling. Also included: population in transit (those on ships, planes, trains and buses) and people in collective dwellings. Brazilians residing abroad, even those who were performing official governmental functions, were not enumerated. The indigenous population was only partially enumerated - only those living in villages in contact with civilization. | 5% sample, but please see "Sampling" | Long-form sample. Systematic sample selected with the same probability inside each census tract. A sample was taken of individual dwellings and of families or individuals in "grupos conviventes" inside collective dwellings with the same sample fraction for tract of that municipality. Each collective dwelling had a separate list form - CD 3 - (Lista de Domicílio Coletivo) to select the families or individuals for the sample. A 25% sample was taken of individual dwellings and of families or individuals in "grupo conviventes". From this original sample, IPUMS drew a systematic sample of every fifth dwelling. **NOTE: Several states totaling approximately one sixth of the national population are missing from the long-form microdata, and are thus not included in the 1960 sample data. These states are Rondônia, Acre, Amazonas, Roraima, Pará, Amapá, Maranhão, Piauí, Espírito Santo, Guanabara, and Santa Catarina. | https://international.ipums.org/international-action/sample_details/country/br#tab_br1991a |
| Greece 2001 | The entire population of the country, including all households and dwellings. | 10% sample | Systematic Sampling (random start, then 1 out of every 2.5 private households from the processed 25% households) by IPUMS. | https://international.ipums.org/international-action/sample_details/country/gr#tab_gr2001a |
| Indonesia 1990 | All population residing in the geographic area of Indonesia regardless of residence status. Homeless, boat people, etc were enumerated. | 0.51% Sample | Data are derived from the sample of census blocks that received the long form questionnaire, stratified by urban-rural status. | https://international.ipums.org/international-action/sample_details/country/id#id1990a |
| Iraq 1997 | All population inside Iraq and Iraqis abroad | 10% Sample | Systematic sample of every tenth private dwelling. Drawn by the IPUMS from 100% microdata. | https://international.ipums.org/international-action/sample_details/country/iq#tab_iq1997a |
| Malawi 2008 | All persons present in Malawi at the time of census. These include foreigners with acknowledged status as refugees, and citizens of Malawi who at the time of census are absent temporarily (less than 6 months). However, diplomatic personnel of the foreign diplomatic and consular representative offices, foreign military personnel and their family members, and the members and representatives of the international organizations and communities located in Malawi are not enumerated | 10% Sample | Systematic sample of every 10th household with a random start, drawn by the IPUMS | https://international.ipums.org/international-action/sample_details/country/mw#mw2008a |
| Malaysia 1980 | The 1980 Population Census included all Malaysians and non- Malaysians who had slept in private or small institutional living quarters during Census Night. However, there were several group of people who did not fall into the above category and for whom special arrangements were made: (a) Persons living in remote and security areas - These include the Orang Asli (Aborigines) in Peninsular Malaysia and other indigenous groups who live deep in the jungles in the interior of Sabah and Sarawak; also persons in security areas were included. The enumeration of such persons was done in a single stage operation. (b) Military, naval and diplomatic personnel and their families and civilians staying outside Malaysia - Since the strategy of enumeration was based on the de facto approach, this group of persons were excluded from the count. Civilians who were temporarily away from the country were also excluded. (c) Resident merchants, seamen and passengers who were at sea on Census night The following were included:- (i) Crews and passengers on coastal ships sailing in Malaysian waters or in Malaysian ports or sailing between Malaysian ports; (ii) Crews and passengers on vessels registered in Malaysia and engaged in regional trade. Crews and passengers on foreign registered ships in port or in Malaysian waters were, however, excluded. (d) Civilian residents who cross a frontier daily to work in another country -This group was included in the census if they were in Malaysia on Census night. (e) Foreign military, naval and diplomatic personnel and their families located in the country -All persons in this category were included in the census with the exception of persons having diplomatic immunity. (f) Transients -Wayfarers, homeless people and travellers were included in the census. Special arrangements were made with the police, railway and marine authorities to enumerate these people. (g) Institutions -Institutions in large hotels, hostels, hospitals and prisons were also included. (h) Army camps and army personnel on patrol -Army personnel were included in the census, through the assistance of the commanding officers of the camps. | 1.67% | Unknown. **Note: The states of Borneo (Sabah and Sarawak) are excluded from the sample. | https://international.ipums.org/international-action/sample_details/country/my#my1980a |
| Pakistan 1973 | The HED sample survey was a second phase of the 1972 Census administered to 300,000 households. The first phase was a full-count census in September 1972 that used a seven-question short form. The HED questionnaire contains two parts. Part I asks questions on housing characteristics and household facilities for both urban and rural areas. Part II asks questions particulars of household member. | 2% Sample | Approximately 24 thousand blocks were selected out of 75 thousand in the country. A sample of households would be taken from each block to yield 300,000 households. Urban households were oversampled relative to rural. Roughly 15% of households do not have a head and appear to be fragments. *NOTE: The sample excludes 4 districts in the North-West Frontier Province: Chitral, Dir, Swat, and Malakand Agency. | https://international.ipums.org/international-action/sample_details/country/pk#tab_pk1973a |
| Philippines 1990 | Filipino nationals regardless of whether they are residing in Philipines at the time of the census and citizens of other countires having their usual residence in the Phillipines or those whose temporary residence will exceed a year from the time of their arrival. | 10% Sample | The sampling rate, or the proportion of households to be selected as samples within each enumeration area (EA), varies from one city /municipality to another. It can be either 100%, 20% or 10% depending on the 1990 expected population of the municipality or city. | https://international.ipums.org/international-action/sample_details/country/ph#tab_ph1990a |
| Romania 2002 | Population did not include Romanian citizens with a legal residence in Romania who left the country more than one year ago, but it included foreign citizens who established their usual residence in Romania more than one year ago. | 10% Sample | Systematic; every tenth household after a random start | https://international.ipums.org/international-action/sample_details/country/ro#ro2002a |
| Sudan 2008 | Residents of Sudan | 16.6% Sample | Long form questionnaire for sedentary households (selected enumeration areas) and a sample of nomad households. | https://international.ipums.org/international-action/sample_details/country/sd#tab_sd2008a |
| Thailand 1980 | All Thai nationals residing in Thailand on the census date; foreign civilians who normally reside in Thailand or who temporarily reside in Thailand 3 months or more before the census date; any individual who has normally resided in Thailand but was away for military training, sailing, or temporarily travelling abroad; and Thai civil/military/diplomatic officers and their families who normally have their offices in foreign countries. | 1% Sample | The sample was obtained by drawing a stratified sample of "Long Form" questionnaires. The strata were Bangkok and the four regions (Central, North, Northeastern, South) of Thailand, cross-classified by municipal and nonmunicipal areas. | https://international.ipums.org/international-action/sample_details/country/th#th1980a |
| United States 1980 | Residents of the 50 states (not the outlying areas). | 5% Sample | 1-in-20 national random sample drawn by the U.S. Census Bureau | https://international.ipums.org/international-action/sample_details/country/us#us1980a |
| Zambia 2010 | Population of Zambia no further description aviabale | 10% Sample | 10 percent sample drawn by Central Statistical Office with Technical support from the US Census Bureau from 100 % Microdata | https://international.ipums.org/international-action/sample_details/country/zm#zm2010a |

**Table S7.** First model: Generalized linear mixed model of woman’s age and her reproductive time span, and presence of any mother in the household, regressing on the woman’s number of children on basis of a Poisson error structure, with sample as random factor.

|  | **Value** | **Std.Error** | **t-value** | **p-value** |
| --- | --- | --- | --- | --- |
| (Intercept) | -0.4062 | 0.0673 | -6.0361 | p < 0.0001 |
| no Mother in HH (ref. Own Mother in HH) | 0.1346 | 0.0027 | 50.6970 | p < 0.0001 |
| Mother in law in HH (ref. Own Mother in HH) | 0.0220 | 0.0031 | 7.1489 | p < 0.0001 |
| Age | 0.0068 | 0.0001 | 46.5537 | p < 0.0001 |
| Reproductive time | 0.1080 | 0.0001 | 769.1488 | p < 0.0001 |
| DF | 2379227 |  |  |  |
|  | (Intercept) | Residual |  |  |
| SAMPLE StdDev: | 0.2600403 | 0.8594551 |  |  |
| Residual deviance: 2289173 on 2379241 degrees of freedom |  |  |  |  |
| AIC: 7166272 |  |  |  |  |

**Table S8.** Second model: Generalized linear mixed model of woman’s age, her reproductive time span, and woman’s education, and presence of any mother in the household, regressing on the woman’s number of children on basis of a Poisson error structure, with sample as random factor.

|  | **Value** | **Std.Error** | **t-value** | **p-value** |
| --- | --- | --- | --- | --- |
| (Intercept) | -0.5188 | 0.0531 | -9.7713 | p < 0.0001 |
| no Mother in HH (ref. Own Mother in HH) | 0.1114 | 0.0026 | 42.7755 | p < 0.0001 |
| Mother in law in HH (ref. Own Mother in HH) | -0.0015 | 0.0030 | -0.4838 | 0.6285 |
| Age | 0.0198 | 0.0002 | 131.6573 | p < 0.0001 |
| Reproductive time | 0.0950 | 0.0001 | 653.6139 | p < 0.0001 |
| primary school completed (ref. less than primary completed) | -0.0958 | 0.0012 | -81.1859 | p < 0.0001 |
| secondary completed (ref. less than primary completed) | -0.2917 | 0.0015 | -198.7490 | p < 0.0001 |
| university completed (ref. less than primary completed) | -0.5560154 | 0.0023426 | -237.3498 | p < 0.0001 |
| DF | 2368034 |  |  |  |
|  | (Intercept) | Residual |  |  |
| SAMPLE StdDev: | 0.2049186 | 0.84099 |  |  |
| Residual deviance: 2090070 on 2368048 degrees of freedom |  |  |  |  |
| AIC: 6943000 |  |  |  |  |

**Table S9.** Third model: Generalized linear mixed model of woman’s age, her reproductive time span, and woman’s education and employment, and presence of any mother in the household, regressing on the woman’s number of children on basis of a Poisson error structure, with sample as random factor.

|  | **Value** | **Std.Error** | **t-value** | **p-value** |
| --- | --- | --- | --- | --- |
| (Intercept) | -0.4901 | 0.0556 | -8.8199 | p < 0.0001 |
| no Mother in HH (ref. Own Mother in HH) | 0.1054 | 0.0026 | 39.8144 | p < 0.0001 |
| Mother in law in HH (ref. Own Mother in HH) | -0.0076 | 0.0031 | -2.4963 | 0.0125 |
| Age | 0.0213 | 0.0002 | 141.0349 | p < 0.0001 |
| Woman’s reproductive time span | 0.0943 | 0.0001 | 647.8666 | p < 0.0001 |
| Woman: primary school completed (ref.: less than primary completed) | -0.0942 | 0.0012 | -79.9444 | p < 0.0001 |
| Woman: secondary completed (ref.: less than primary completed) | -0.2626 | 0.0015 | -178.4965 | p < 0.0001 |
| Woman: university completed (ref.: less than primary completed) | -0.4939 | 0.0024 | -209.3824 | p < 0.0001 |
| Woman: employed (ref.: not employed) | -0.1878 | 0.0010 | -191.7922 | p < 0.0001 |
| DF | 2343375 |  |  |  |
|  | (Intercept) | Residual |  |  |
| SAMPLE StdDev: | 0.2072008 | 0.8372617 |  |  |
| Residual deviance: 2031030 on 2343388 degrees of freedom |  |  |  |  |
| AIC: 6830540 |  |  |  |  |

**Table S10.** Fourth model: Generalized linear mixed model of woman’s age, her reproductive time span, woman’s and her spouse’s education, and woman’s employment, and presence of any mother in the household, regressing on the woman’s number of children on basis of a Poisson error structure, with sample as random factor.

|  | **Value** | **Std.Error** | **t-value** | **p-value** |
| --- | --- | --- | --- | --- |
| (Intercept) | -0.4846 | 0.0549 | -8.8237 | p < 0.0001 |
| no Mother in HH (ref. Own Mother in HH) | 0.1053 | 0.0026 | 39.8556 | p < 0.0001 |
| Mother in law in HH (ref. Own Mother in HH) | -0.0057 | 0.0031 | -1.8607 | 0.0628 |
| Age | 0.0221 | 0.0002 | 146.0422 | p < 0.0001 |
| Woman’s reproductive time span | 0.0935 | 0.0001 | 640.7982 | p < 0.0001 |
| Woman: primary school completed (ref.: less than primary completed) | -0.0673 | 0.0013 | -51.6214 | p < 0.0001 |
| Woman: secondary completed (ref.: less than primary completed) | -0.2023 | 0.0017 | -118.2312 | p < 0.0001 |
| Woman: university completed (ref.: less than primary completed) | -0.3765 | 0.0027 | -138.1740 | p < 0.0001 |
| Woman: employed (ref.: not employed) | -0.1893 | 0.0010 | -193.3801 | p < 0.0001 |
| Spouse: primary school completed (ref.: less than primary completed) | -0.0407 | 0.0013 | -31.8643 | p < 0.0001 |
| Spouse: secondary completed (ref.: less than primary completed) | -0.0919 | 0.0016 | -57.6869 | p < 0.0001 |
| Spouse: university completed (ref.: less than primary completed) | -0.1948 | 0.0023 | -82.9654 | p < 0.0001 |
| DF | 2329895 |  |  |  |
|  | (Intercept) | Residual |  |  |
| SAMPLE StdDev: | 0.2048 | 0.8345 |  |  |
| Residual deviance: 2004484 on 2329908 degrees of freedom |  |  |  |  |
| AIC: 6774205 |  |  |  |  |

**Table S11.** Fifth model: Generalized linear mixed model of woman’s age, her reproductive time span, woman’s and her spouse’s education and employment, and presence of any mother in the household, regressing on the woman’s number of children on basis of a Poisson error structure, with sample as random factor.

|  | **Value** | **Std.Error** | **t-value** | **p-value** |
| --- | --- | --- | --- | --- |
| (Intercept) | -0.5080 | 0.0548 | -9.2622 | p < 0.0001 |
| no Mother in HH (ref. Own Mother in HH) | 0.1037 | 0.0026 | 39.2065 | p < 0.0001 |
| Mother in law in HH (ref. Own Mother in HH) | -0.0061 | 0.0031 | -1.9976 | 0.0458 |
| Age | 0.0221 | 0.0002 | 145.8538 | p < 0.0001 |
| Woman’s reproductive time span | 0.0935 | 0.0001 | 640.6359 | p < 0.0001 |
| Woman: primary school completed (ref.: less than primary completed) | -0.0674 | 0.0013 | -51.7111 | p < 0.0001 |
| Woman: secondary completed (ref.: less than primary completed) | -0.2026 | 0.0017 | -118.3936 | p < 0.0001 |
| Woman: university completed (ref.: less than primary completed) | -0.3764 | 0.0027 | -138.1172 | p < 0.0001 |
| Woman: employed (ref.: not employed) | -0.1909 | 0.0010 | -194.3502 | p < 0.0001 |
| Spouse: primary school completed (ref.: less than primary completed) | -0.0407 | 0.0013 | -31.9195 | p < 0.0001 |
| Spouse: secondary completed (ref.: less than primary completed) | -0.0926 | 0.0016 | -58.1312 | p < 0.0001 |
| Spouse: university completed (ref.: less than primary completed) | -0.1964 | 0.0023 | -83.5785 | p < 0.0001 |
| Spouse: employed (ref.: not empolyed) | 0.0292 | 0.0014 | 20.2285 | p < 0.0001 |
| DF | 2329566 |  |  |  |
|  | (Intercept) | Residual |  |  |
| SAMPLE StdDev: | 0.2044694 | 0.8345547 |  |  |
| Residual deviance: 2003987 on 2329579 degrees of freedom |  |  |  |  |
| AIC: 6773069 |  |  |  |  |

**Table S12**. Separate generalized linear mixed model for each census of woman’s age, her reproductive time span, woman’s and her spouse’s education and employment, living area, and presence of any mother in the household, regressing on the woman’s number of children on basis of a Poisson error structure, with sample as random factor.

| **Argentinia** |  |  |  |  |
| --- | --- | --- | --- | --- |
|  | **Estimate** | **Std. Error** | **z value** | **P** |
| (Intercept) | 0.0741 | 0.0266 | 2.7860 | 0.00534 |
| no Mother in HH (ref. Own Mother in HH) | 0.1150 | 0.0122 | 9.4000 | p < 0.0001 |
| Mother in law in HH (ref. Own Mother in HH) | 0.0421 | 0.0152 | 2.7660 | 0.00568 |
| Age | 0.0147 | 0.0009 | 16.7690 | p < 0.0001 |
| Reproductive time | 0.0789 | 0.0008 | 98.2740 | p < 0.0001 |
| primary school completed (ref. less than primary completed) | -0.1386 | 0.0110 | -12.5670 | p < 0.0001 |
| secondary completed (ref. less than primary completed) | -0.3111 | 0.0121 | -25.7300 | p < 0.0001 |
| university completed (ref. less than primary completed) | -0.4864 | 0.0183 | -26.6010 | p < 0.0001 |
| Spouse primary school completed (ref. less than primary completed) | -0.1014 | 0.0104 | -9.7560 | p < 0.0001 |
| Spouse secondary completed (ref. less than primary completed) | -0.1969 | 0.0117 | -16.8910 | p < 0.0001 |
| Spouse university completed (ref. less than primary completed) | -0.2679 | 0.0173 | -15.4620 | p < 0.0001 |
| Employeed yes (ref. No) | -0.2016 | 0.0061 | -32.8570 | p < 0.0001 |
| Spouse employeed yes (ref. No) | -0.0350 | 0.0066 | -5.2930 | p < 0.0001 |
| Urban (ref. Rural) | -0.0867 | 0.0081 | -10.7220 | p < 0.0001 |
| Residual deviance: 63074 on 86652 degrees of freedom |  |  |  |  |
| AIC: 250706 |  |  |  |  |
|  |  |  |  |  |
| **Brazil** |  |  |  |  |
|  | **Estimate** | **Std. Error** | **z value** | **P** |
| (Intercept) | -0.4016 | 0.0118 | -34.1230 | p < 0.0001 |
| no Mother in HH (ref. Own Mother in HH) | 0.0512 | 0.0056 | 9.1040 | p < 0.0001 |
| Mother in law in HH (ref. Own Mother in HH) | -0.0871 | 0.0077 | -11.2800 | p < 0.0001 |
| Age | 0.0220 | 0.0003 | 62.9040 | p < 0.0001 |
| Reproductive time | 0.0934 | 0.0004 | 257.6600 | p < 0.0001 |
| primary school completed (ref. less than primary completed) | -0.1261 | 0.0030 | -42.4920 | p < 0.0001 |
| secondary completed (ref. less than primary completed) | -0.2048 | 0.0043 | -47.2250 | p < 0.0001 |
| university completed (ref. less than primary completed) | -0.2779 | 0.0088 | -31.4570 | p < 0.0001 |
| Spouse primary school completed (ref. less than primary completed) | -0.1186 | 0.0030 | -39.3940 | p < 0.0001 |
| Spouse secondary completed (ref. less than primary completed) | -0.1500 | 0.0042 | -35.5510 | p < 0.0001 |
| Spouse university completed (ref. less than primary completed) | -0.1827 | 0.0079 | -22.9860 | p < 0.0001 |
| Employeed yes (ref. No) | -0.1626 | 0.0027 | -60.3210 | p < 0.0001 |
| Spouse employeed yes (ref. No) | -0.0236 | 0.0074 | -3.1880 | 0.00143 |
| Urban (ref. Rural) | -0.0913 | 0.0026 | -35.0350 | p < 0.0001 |
| Residual deviance: 353546 on 465471 degrees of freedom |  |  |  |  |
| AIC: 1329528 |  |  |  |  |
|  |  |  |  |  |
| **Greece** |  |  |  |  |
|  | **Estimate** | **Std. Error** | **z value** | **P** |
| (Intercept) | -0.4836 | 0.0635 | -7.6100 | p < 0.0001 |
| no Mother in HH (ref. Own Mother in HH) | 0.0726 | 0.0366 | 1.9870 | 0.047 |
| Mother in law in HH (ref. Own Mother in HH) | 0.1850 | 0.0398 | 4.6470 | p < 0.0001 |
| Age | 0.0139 | 0.0019 | 7.2590 | p < 0.0001 |
| Reproductive time | 0.0983 | 0.0018 | 55.0960 | p < 0.0001 |
| primary school completed (ref. less than primary completed) | -0.2495 | 0.0401 | -6.2190 | p < 0.0001 |
| secondary completed (ref. less than primary completed) | -0.3047 | 0.0412 | -7.4040 | p < 0.0001 |
| university completed (ref. less than primary completed) | -0.4025 | 0.0441 | -9.1210 | p < 0.0001 |
| Spouse primary school completed (ref. less than primary completed) | -0.1018 | 0.0413 | -2.4620 | 0.0138 |
| Spouse secondary completed (ref. less than primary completed) | -0.1861 | 0.0424 | -4.3840 | p < 0.0001 |
| Spouse university completed (ref. less than primary completed) | -0.2578 | 0.0456 | -5.6540 | p < 0.0001 |
| Employeed yes (ref. No) | -0.1819 | 0.0118 | -15.4770 | p < 0.0001 |
| Spouse employeed yes (ref. No) | 0.0522 | 0.0211 | 2.4730 | 0.0134 |
| Residual deviance: 19791 on 28291 degrees of freedom |  |  |  |  |
| AIC: 67369 |  |  |  |  |
|  |  |  |  |  |
| **Indonesia** |  |  |  |  |
|  | **Estimate** | **Std. Error** | **z value** | **P** |
| (Intercept) | -1.144804 | 0.036102 | -31.71 | p < 0.0001 |
| no Mother in HH (ref. Own Mother in HH) | 0.273969 | 0.014183 | 19.316 | p < 0.0001 |
| Mother in law in HH (ref. Own Mother in HH) | 0.221116 | 0.016301 | 13.565 | p < 0.0001 |
| Age | 0.032667 | 0.001234 | 26.468 | p < 0.0001 |
| Reproductive time | 0.088063 | 0.001118 | 78.764 | p < 0.0001 |
| primary school completed (ref. less than primary completed) | -0.055594 | 0.007462 | -7.45 | p < 0.0001 |
| secondary completed (ref. less than primary completed) | -0.111962 | 0.014408 | -7.771 | p < 0.0001 |
| university completed (ref. less than primary completed) | -0.306206 | 0.0505 | -6.064 | p < 0.0001 |
| Spouse primary school completed (ref. less than primary completed) | -0.032434 | 0.007535 | -4.304 | p < 0.0001 |
| Spouse secondary completed (ref. less than primary completed) | -0.026201 | 0.012165 | -2.154 | 0.03125 |
| Spouse university completed (ref. less than primary completed) | 0.003546 | 0.033139 | 0.107 | 0.91477 |
| Employeed yes (ref. No) | -0.067788 | 0.006485 | -10.453 | p < 0.0001 |
| Spouse employeed yes (ref. No) | 0.060224 | 0.023928 | 2.517 | 0.01184 |
| Urban (ref. Rural) | 0.021176 | 0.007411 | 2.857 | 0.00427 |
| Residual deviance: 40638 on 60577 degrees of freedom |  |  |  |  |
| AIC:169502 |  |  |  |  |
|  |  |  |  |  |
| **Malwai** |  |  |  |  |
|  | **Estimate** | **Std. Error** | **z value** | **P** |
| (Intercept) | -1.1579407 | 0.0352589 | -32.841 | p < 0.0001 |
| no Mother in HH (ref. Own Mother in HH) | 0.2003857 | 0.028977 | 6.915 | p < 0.0001 |
| Mother in law in HH (ref. Own Mother in HH) | 0.0592318 | 0.0325734 | 1.818 | 0.069 |
| Age | 0.0551553 | 0.0010114 | 54.536 | p < 0.0001 |
| Reproductive time | 0.0667333 | 0.0009948 | 67.084 | p < 0.0001 |
| primary school completed (ref. less than primary completed) | -0.0863595 | 0.006064 | -14.241 | p < 0.0001 |
| secondary completed (ref. less than primary completed) | -0.2640857 | 0.0126113 | -20.94 | p < 0.0001 |
| university completed (ref. less than primary completed) | -0.5912492 | 0.1063103 | -5.562 | p < 0.0001 |
| Spouse primary school completed (ref. less than primary completed) | -0.0334628 | 0.0053742 | -6.227 | p < 0.0001 |
| Spouse secondary completed (ref. less than primary completed) | -0.0705236 | 0.0082615 | -8.536 | p < 0.0001 |
| Spouse university completed (ref. less than primary completed) | -0.1969615 | 0.0489143 | -4.027 | p < 0.0001 |
| Employeed yes (ref. No) | -0.0076405 | 0.0049881 | -1.532 | 0.126 |
| Spouse employeed yes (ref. No) | 0.0259267 | 0.0065489 | 3.959 | p < 0.0001 |
| Urban (ref. Rural) | -0.056847 | 0.0071607 | -7.939 | p < 0.0001 |
| Residual deviance: 62151 on 91998 degrees of freedom |  |  |  |  |
| AIC: 278846 |  |  |  |  |
|  |  |  |  |  |
| **Iraq** |  |  |  |  |
|  | **Estimate** | **Std. Error** | **z value** | **P** |
| (Intercept) | -0.5199 | 0.0273 | -19.0530 | p < 0.0001 |
| no Mother in HH (ref. Own Mother in HH) | 0.2099 | 0.0225 | 9.3260 | p < 0.0001 |
| Mother in law in HH (ref. Own Mother in HH) | 0.1216 | 0.0226 | 5.3930 | p < 0.0001 |
| Age | 0.0193 | 0.0006 | 29.9720 | p < 0.0001 |
| Reproductive time | 0.1131 | 0.0006 | 178.5900 | p < 0.0001 |
| primary school completed (ref. less than primary completed) | -0.0110 | 0.0048 | -2.2880 | 0.0222 |
| secondary completed (ref. less than primary completed) | -0.1232 | 0.0091 | -13.5240 | p < 0.0001 |
| university completed (ref. less than primary completed) | -0.2263 | 0.0170 | -13.3170 | p < 0.0001 |
| Spouse primary school completed (ref. less than primary completed) | 0.0097 | 0.0053 | 1.8130 | 0.0698 |
| Spouse secondary completed (ref. less than primary completed) | 0.0274 | 0.0066 | 4.1300 | p < 0.0001 |
| Spouse university completed (ref. less than primary completed) | 0.0106 | 0.0094 | 1.1180 | 0.2634 |
| Employeed yes (ref. No) | -0.0417 | 0.0096 | -4.3290 | p < 0.0001 |
| Spouse employeed yes (ref. No) | 0.0635 | 0.0066 | 9.5740 | p < 0.0001 |
| Urban (ref. Rural) | -0.0856 | 0.0043 | -19.7610 | p < 0.0001 |
| Residual deviance: 76419 on 102884 degrees of freedom |  |  |  |  |
| AIC: 321667 |  |  |  |  |
|  |  |  |  |  |
| **Malaysia** |  |  |  |  |
|  | **Estimate** | **Std. Error** | **z value** | **P** |
| (Intercept) | -0.2780 | 0.0903 | -3.0810 | 0.002065 |
| no Mother in HH (ref. Own Mother in HH) | 0.0840 | 0.0385 | 2.1820 | 0.029146 |
| Mother in law in HH (ref. Own Mother in HH) | 0.0791 | 0.0415 | 1.9040 | 0.056968 |
| Age | 0.0103 | 0.0028 | 3.7180 | p < 0.0001 |
| Reproductive time | 0.1067 | 0.0026 | 41.3350 | p < 0.0001 |
| primary school completed (ref. less than primary completed) | -0.0685 | 0.0172 | -3.9860 | 6.71E-05 |
| secondary completed (ref. less than primary completed) | -0.2648 | 0.0887 | -2.9840 | 0.002841 |
| university completed (ref. less than primary completed) | -0.0357 | 0.0964 | -0.3710 | 0.711004 |
| Spouse primary school completed (ref. less than primary completed) | -0.0322 | 0.0196 | -1.6420 | 0.100577 |
| Spouse secondary completed (ref. less than primary completed) | -0.1854 | 0.0661 | -2.8030 | 0.005057 |
| Spouse university completed (ref. less than primary completed) | -0.2515 | 0.0654 | -3.8480 | 0.000119 |
| Employeed yes (ref. No) | -0.1047 | 0.0171 | -6.1410 | p < 0.0001 |
| Spouse employeed yes (ref. No) | 0.0938 | 0.0568 | 1.6520 | 0.098592 |
| Urban (ref. Rural) | 0.0100 | 0.0162 | 0.6150 | 0.538538 |
| Residual deviance: 5769.1 on 8369 degrees of freedom |  |  |  |  |
| AIC: 25025 |  |  |  |  |
|  |  |  |  |  |
| **Pakistan** |  |  |  |  |
|  | **Estimate** | **Std. Error** | **z value** | **P** |
| (Intercept) | -0.8540 | 0.0407 | -20.9710 | p < 0.0001 |
| no Mother in HH (ref. Own Mother in HH) | 0.1600 | 0.0308 | 5.1920 | p < 0.0001 |
| Mother in law in HH (ref. Own Mother in HH) | 0.0150 | 0.0310 | 0.4830 | 0.629351 |
| Age | 0.0091 | 0.0013 | 7.1340 | p < 0.0001 |
| Reproductive time | 0.1414 | 0.0011 | 125.2920 | p < 0.0001 |
| primary school completed (ref. less than primary completed) | 0.0253 | 0.0102 | 2.4820 | 0.013053 |
| secondary completed (ref. less than primary completed) | -0.0582 | 0.0304 | -1.9120 | 0.055854 |
| university completed (ref. less than primary completed) | -0.1349 | 0.0427 | -3.1610 | 0.001573 |
| Spouse primary school completed (ref. less than primary completed) | 0.0317 | 0.0063 | 5.0200 | p < 0.0001 |
| Spouse secondary completed (ref. less than primary completed) | 0.0561 | 0.0169 | 3.3210 | 0.000898 |
| Spouse university completed (ref. less than primary completed) | 0.0243 | 0.0169 | 1.4380 | 0.150472 |
| Employeed yes (ref. No) | -0.0253 | 0.0151 | -1.6740 | 0.094064 |
| Spouse employeed yes (ref. No) | 0.1176 | 0.0116 | 10.1400 | p < 0.0001 |
| Urban (ref. Rural) | 0.1136 | 0.0058 | 19.5040 | p < 0.0001 |
| Residual deviance: 63973 on 66092 degrees of freedom |  |  |  |  |
| AIC: 1477.6 |  |  |  |  |
|  |  |  |  |  |
| **Romania** |  |  |  |  |
|  | **Estimate** | **Std. Error** | **z value** | **P** |
| (Intercept) | -0.1318 | 0.0307 | -4.2950 | p < 0.0001 |
| no Mother in HH (ref. Own Mother in HH) | 0.1117 | 0.0106 | 10.5550 | p < 0.0001 |
| Mother in law in HH (ref. Own Mother in HH) | 0.0409 | 0.0120 | 3.4180 | 0.00063 |
| Age | 0.0138 | 0.0012 | 11.9820 | p < 0.0001 |
| Reproductive time | 0.0806 | 0.0011 | 71.9120 | p < 0.0001 |
| primary school completed (ref. less than primary completed) | -0.1972 | 0.0144 | -13.7060 | p < 0.0001 |
| secondary completed (ref. less than primary completed) | -0.3654 | 0.0148 | -24.6630 | p < 0.0001 |
| university completed (ref. less than primary completed) | -0.5395 | 0.0227 | -23.7430 | p < 0.0001 |
| Spouse primary school completed (ref. less than primary completed) | -0.1690 | 0.0168 | -10.0740 | p < 0.0001 |
| Spouse secondary completed (ref. less than primary completed) | -0.2753 | 0.0167 | -16.5080 | p < 0.0001 |
| Spouse university completed (ref. less than primary completed) | -0.3794 | 0.0233 | -16.2590 | p < 0.0001 |
| Employeed yes (ref. No) | -0.1452 | 0.0060 | -24.1910 | p < 0.0001 |
| Spouse employeed yes (ref. No) | -0.0114 | 0.0077 | -1.4860 | 0.13733 |
| Urban (ref. Rural) | -0.2362 | 0.0064 | -37.0200 | p < 0.0001 |
| Residual deviance: 61353 on 712652 degrees of freedom |  |  |  |  |
| AIC: 249563 |  |  |  |  |
|  |  |  |  |  |
| **USA** |  |  |  |  |
|  | **Estimate** | **Std. Error** | **z value** | **P** |
| (Intercept) | -0.1611453 | 0.0166989 | -9.65 | p < 0.0001 |
| no Mother in HH (ref. Own Mother in HH) | 0.0324932 | 0.0092153 | 3.526 | 0.000422 |
| Mother in law in HH (ref. Own Mother in HH) | 0.0083211 | 0.0128286 | 0.649 | 0.516573 |
| Age | 0.0137688 | 0.0004538 | 30.338 | p < 0.0001 |
| Reproductive time | 0.0998968 | 0.0004211 | 237.252 | p < 0.0001 |
| primary school completed (ref. less than primary completed) | -0.0800137 | 0.0094929 | -8.429 | p < 0.0001 |
| secondary completed (ref. less than primary completed) | -0.24678 | 0.0094769 | -26.04 | p < 0.0001 |
| university completed (ref. less than primary completed) | -0.4704548 | 0.0101897 | -46.17 | p < 0.0001 |
| Spouse primary school completed (ref. less than primary completed) | -0.111054 | 0.0088232 | -12.587 | p < 0.0001 |
| Spouse secondary completed (ref. less than primary completed) | -0.2016579 | 0.0087715 | -22.99 | p < 0.0001 |
| Spouse university completed (ref. less than primary completed) | -0.3177789 | 0.0091813 | -34.612 | p < 0.0001 |
| Employeed yes (ref. No) | -0.405559 | 0.0021087 | -192.33 | p < 0.0001 |
| Spouse employeed yes (ref. No) | -0.0358765 | 0.003727 | -9.626 | p < 0.0001 |
| Residual deviance: 214749 on 187042 degrees of freedom |  |  |  |  |
| AIC: 604019 |  |  |  |  |
|  |  |  |  |  |
| **Sudan** |  |  |  |  |
|  | **Estimate** | **Std. Error** | **z value** | **P** |
| (Intercept) | -1.3182 | 0.0242 | -54.5070 | p < 0.0001 |
| no Mother in HH (ref. Own Mother in HH) | 0.3919 | 0.0209 | 18.7260 | p < 0.0001 |
| Mother in law in HH (ref. Own Mother in HH) | 0.2246 | 0.0219 | 10.2360 | p < 0.0001 |
| Age | 0.0443 | 0.0006 | 77.0580 | p < 0.0001 |
| Reproductive time | 0.0803 | 0.0005 | 151.8920 | p < 0.0001 |
| primary school completed (ref. less than primary completed) | -0.1185 | 0.0086 | -13.7620 | p < 0.0001 |
| secondary completed (ref. less than primary completed) | -0.2306 | 0.0357 | -6.4560 | p < 0.0001 |
| university completed (ref. less than primary completed) | -0.5334 | 0.0220 | -24.2250 | p < 0.0001 |
| Spouse primary school completed (ref. less than primary completed) | 0.0252 | 0.0073 | 3.4760 | 0.00051 |
| Spouse secondary completed (ref. less than primary completed) | -0.0157 | 0.0266 | -0.5910 | 0.554437 |
| Spouse university completed (ref. less than primary completed) | -0.1133 | 0.0183 | -6.1860 | p < 0.0001 |
| Employeed yes (ref. No) | 0.0155 | 0.0045 | 3.4780 | 0.000504 |
| Spouse employeed yes (ref. No) | 0.0625 | 0.0049 | 12.7020 | p < 0.0001 |
| Urban (ref. Rural) | 0.0183 | 0.0065 | 2.8270 | 0.004695 |
| Residual deviance: 245081 on 105167 degrees of freedom |  |  |  |  |
| AIC: 1080262 |  |  |  |  |
|  |  |  |  |  |
| **Philipines** |  |  |  |  |
|  | **Estimate** | **Std. Error** | **z value** | **P** |
| (Intercept) | -0.3202 | 0.0112 | -28.6350 | p < 0.0001 |
| no Mother in HH (ref. Own Mother in HH) | 0.1886 | 0.0063 | 30.0850 | p < 0.0001 |
| Mother in law in HH (ref. Own Mother in HH) | 0.0190 | 0.0078 | 2.4350 | 0.0149 |
| Age | 0.0152 | 0.0004 | 36.7230 | p < 0.0001 |
| Reproductive time | 0.0907 | 0.0004 | 224.8720 | p < 0.0001 |
| primary school completed (ref. less than primary completed) | -0.0130 | 0.0033 | -3.9280 | 8.55E-05 |
| secondary completed (ref. less than primary completed) | -0.0679 | 0.0041 | -16.4200 | p < 0.0001 |
| university completed (ref. less than primary completed) | -0.1342 | 0.0065 | -20.5820 | p < 0.0001 |
| Spouse primary school completed (ref. less than primary completed) | -0.0130 | 0.0032 | -4.0630 | p < 0.0001 |
| Spouse secondary completed (ref. less than primary completed) | -0.0458 | 0.0039 | -11.7180 | p < 0.0001 |
| Spouse university completed (ref. less than primary completed) | -0.0863 | 0.0066 | -13.0240 | p < 0.0001 |
| Employeed yes (ref. No) | -0.0595 | 0.0028 | -21.1310 | p < 0.0001 |
| Spouse employeed yes (ref. No) | 0.0450 | 0.0035 | 12.9870 | p < 0.0001 |
| Urban (ref. Rural) | -0.0443 | 0.0025 | -17.8750 | p < 0.0001 |
| Residual deviance: 582245 on 348336 degrees of freedom |  |  |  |  |
| AIC: 1826027 |  |  |  |  |
|  |  |  |  |  |
|  |  |  |  |  |
|  |  |  |  |  |
| **Zambia** |  |  |  |  |
|  | **Estimate** | **Std. Error** | **z value** | **P** |
| (Intercept) | -0.7306 | 0.0339 | -21.5700 | p < 0.0001 |
| no Mother in HH (ref. Own Mother in HH) | 0.0554 | 0.0258 | 2.1470 | 0.0318 |
| Mother in law in HH (ref. Own Mother in HH) | -0.0607 | 0.0305 | -1.9900 | 0.0466 |
| Age | 0.0454 | 0.0010 | 43.4820 | p < 0.0001 |
| Reproductive time | 0.0698 | 0.0010 | 70.2310 | p < 0.0001 |
| primary school completed (ref. less than primary completed) | -0.0747 | 0.0060 | -12.3890 | p < 0.0001 |
| secondary completed (ref. less than primary completed) | -0.3295 | 0.0129 | -25.4750 | p < 0.0001 |
| university completed (ref. less than primary completed) | -0.6424 | 0.0778 | -8.2570 | p < 0.0001 |
| Spouse primary school completed (ref. less than primary completed) | -0.0136 | 0.0065 | -2.1120 | 0.0346 |
| Spouse secondary completed (ref. less than primary completed) | -0.0987 | 0.0098 | -10.0750 | p < 0.0001 |
| Spouse university completed (ref. less than primary completed) | -0.2558 | 0.0423 | -6.0540 | p < 0.0001 |
| Employeed yes (ref. No) | 0.0324 | 0.0055 | 5.8740 | p < 0.0001 |
| Spouse employeed yes (ref. No) | 0.0411 | 0.0082 | 5.0090 | p < 0.0001 |
| Residual deviance: 58889 on 64764 degrees of freedom |  |  |  |  |
| AIC: 211322 |  |  |  |  |
|  |  |  |  |  |
| **Thailand** |  |  |  |  |
|  | **Estimate** | **Std. Error** | **z value** | **P** |
| (Intercept) | -0.5124 | 0.0397 | -12.8940 | p < 0.0001 |
| no Mother in HH (ref. Own Mother in HH) | 0.1796 | 0.0159 | 11.2920 | p < 0.0001 |
| Mother in law in HH (ref. Own Mother in HH) | 0.1122 | 0.0204 | 5.4920 | p < 0.0001 |
| Age | 0.0137 | 0.0018 | 7.6370 | p < 0.0001 |
| Reproductive time | 0.1029 | 0.0018 | 56.7480 | p < 0.0001 |
| primary school completed (ref. less than primary completed) | -0.1004 | 0.0228 | -4.4120 | p < 0.0001 |
| secondary completed (ref. less than primary completed) | -0.1857 | 0.0389 | -4.7770 | p < 0.0001 |
| university completed (ref. less than primary completed) | -0.4415 | 0.1030 | -4.2880 | p < 0.0001 |
| Spouse primary school completed (ref. less than primary completed) | -0.0356 | 0.0170 | -2.0860 | 0.03697 |
| Spouse secondary completed (ref. less than primary completed) | -0.0843 | 0.0305 | -2.7660 | 0.00568 |
| Spouse university completed (ref. less than primary completed) | -0.0555 | 0.0711 | -0.7810 | 0.4349 |
| Urban (ref. Rural) | -0.0839 | 0.0161 | -5.1960 | p < 0.0001 |
| Residual deviance: 16723 on 23719 degrees of freedom |  |  |  |  |
| AIC: 68980 |  |  |  |  |

**Table S13.** Generalized linear mixed model of woman’s age, woman’s and her spouse’s education and employment, and living area regressing on presence of (a) mother’s own mother and (b) spouse’s mother in the household on basis of a Poisson error structure, with sample as random factor.

| 1. **OWN Mother in HH** |  |  |  |  |
| --- | --- | --- | --- | --- |
|  |  |  |  |  |
|  | **Value** | **Std.Error** | **t-value** | **P** |
| (Intercept) | -0.5669 | 0.2712 | -2.0901 | 0.0366 |
| Spouse primary school completed (ref. less than primary completed) | 0.1312 | 0.0114 | 11.4923 | p < 0.0001 |
| Spouse secondary completed (ref. less than primary completed) | 0.1165 | 0.0136 | 8.5372 | p < 0.0001 |
| Spouse university completed (ref. less than primary completed) | -0.0123 | 0.0211 | -0.5827 | 0.5601 |
| primary school completed (ref. less than primary completed) | 0.2793 | 0.0117 | 23.9199 | p < 0.0001 |
| secondary completed (ref. less than primary completed) | 0.6882 | 0.0140 | 49.1691 | p < 0.0001 |
| university completed (ref. less than primary completed) | 1.1148 | 0.0207 | 53.9052 | p < 0.0001 |
| Age | -0.1033 | 0.0009 | -109.5860 | p < 0.0001 |
| Urban (ref. Rural) | 0.0978 | 0.0091 | 10.7231 | p < 0.0001 |
| Employeed yes (ref. No) | 0.1205 | 0.0087 | 13.9096 | p < 0.0001 |
| Spouse employeed yes (ref. No) | -0.5212 | 0.0118 | -44.3233 | p < 0.0001 |
| DF | 1413108 |  |  |  |
|  | (Intercept) | Residual |  |  |
| SAMPLE StdDev: | 0.8536185 | 1.003908 |  |  |
|  |  |  |  |  |
| 1. **SPOUSE MOTHER IN HH** |  |  |  |  |
|  |  |  |  |  |
|  | **Value** | **Std.Error** | **t-value** | **P** |
| (Intercept) | 1.1973 | 0.3495 | 3.4256 | 0.0006 |
| Spouse primary school completed (ref. less than primary completed) | 0.2578 | 0.0076 | 33.7978 | p < 0.0001 |
| Spouse secondary completed (ref. less than primary completed) | 0.3739 | 0.0097 | 38.5889 | p < 0.0001 |
| Spouse university completed (ref. less than primary completed) | 0.3060 | 0.0151 | 20.2706 | p < 0.0001 |
| primary school completed (ref. less than primary completed) | 0.0437 | 0.0078 | 5.5994 | p < 0.0001 |
| secondary completed (ref. less than primary completed) | 0.1283 | 0.0104 | 12.3471 | p < 0.0001 |
| university completed (ref. less than primary completed) | 0.2990 | 0.0170 | 17.5781 | p < 0.0001 |
| Age | -0.1104 | 0.0007 | -168.5671 | p < 0.0001 |
| Urban (ref. Rural) | -0.1459 | 0.0062 | -23.7177 | p < 0.0001 |
| Employeed yes (ref. No) | 0.0517 | 0.0069 | 7.4759 | p < 0.0001 |
| Spouse employeed yes (ref. No) | -0.5027 | 0.0081 | -61.9696 | p < 0.0001 |
| DF | 1541176 |  |  |  |
| Sample | (Intercept) | Residual |  |  |
| StdDev: | 1.103771 | 1.004647 |  |  |
